# Imprintability of Newly Hatched Domestic Chicks on an Artificial Object: A Novel High Time-Resolution Apparatus Based on a Running Disc

**DOI:** 10.3389/fphys.2022.822638

**Published:** 2022-03-11

**Authors:** Naoya Aoki, Chihiro Mori, Toshiyuki Fujita, Shouta Serizawa, Shinji Yamaguchi, Toshiya Matsushima, Koichi J. Homma

**Affiliations:** ^1^Department of Molecular Biology, Faculty of Pharmaceutical Sciences, Teikyo University, Tokyo, Japan; ^2^Department of Biological Sciences, Faculty of Pharmaceutical Sciences, Teikyo University, Tokyo, Japan; ^3^Department of Biology, Faculty of Science, Hokkaido University, Hokkaido, Japan

**Keywords:** filial imprinting, learning process, *Gallus gallus domesticus*, domestic chicks, development, sensitive period, critical period

## Abstract

In filial imprinting, newly hatched chicks repeatedly approach a conspicuous object nearby and memorize it, even though it is an artificial object instead of their mother hen. Imprinting on an artificial object in a laboratory setting has a clear sensitive period from post hatch days 1–3 in the case of domestic chicks. However, the establishment of imprintability are difficult to investigate because of the limitations of the behavioral apparatus. In this study, we developed a novel behavioral apparatus, based on a running disc, to investigate the learning processes of imprinting in newly hatched domestic chicks. In the apparatus, the chick repeatedly approaches the imprinting object on the disc. The apparatus sends a transistor-transistor-logic signal every 1/10 turn of the disc to a personal computer through a data acquisition system following the chick’s approach to the imprinting object on the monitor. The imprinting training and tests were designed to define the three learning processes in imprinting. The first process is the one in which chicks spontaneously approach the moving object. The second is an acquired process in which chicks approach an object even when it is static. In the third process, chicks discriminate between the differently colored imprinting object and the control object in the preference test. Using the apparatus, the difference in the chicks’ behavior during or after the sensitive period was examined. During the sensitive period, the chicks at post hatch hour 12 and 18 developed the first imprinting training process. The chicks at post hatch hour 24 maintained learning until the second process. The chicks at post hatch hour 30 reached the discrimination process in the test. After the sensitive period, the chicks reared in darkness until post hatch day 4 exhibited poor first learning process in the training. Thus, this apparatus will be useful for the detection of behavioral changes during neuronal development and learning processes.

## Introduction

Filial imprinting occurs in the early stages of precocial birds’ lives (Spalding, 1873; Lorenz, 1935). Filial imprinting of domestic chicks is a useful model for early learning (Horn, 1986; Matsushima et al., 2003; McCabe, 2019). During imprinting, newly hatched chicks and ducklings repeatedly approach a conspicuous object nearby and then develop a preference toward it (Bateson, 1964, 1966). The learning processes of imprinting have been investigated in a laboratory setting since the 1960s (Bateson, 1966; Horn, 1998). In these studies, the neuronal and molecular mechanisms of imprinting have been investigated extensively (Horn, 1998, 2004; Yamaguchi et al., 2012; Solomonia and McCabe, 2015; Aoki et al., 2018, 2020). Additionally, several genes involved in memory formation during imprinting have been detected using comprehensive gene screening strategies (Yamaguchi et al., 2008, 2011a,2011b,^2012^).

Although newly hatched chicks spontaneously explore static objects (Bolhuis, 1991; Dharmaretnam and Andrew, 1994; Vallortigara and Andrew, 1994; Versace et al., 2016), the chicks are strongly predisposed to approach conspicuous moving objects (Bateson, 1966; Bolhuis, 1991; Salvatierra et al., 1994; Maekawa et al., 2006; McCabe, 2013; Vallortigara and Versace, 2018). A runway-type apparatus was developed to investigate this innate predisposition. The chicks’ motivation to approach the object depended on its characteristics; for example, color, size, shape, movement, and sound. Particularly, movement and sound were the most effective motivators (Collias and Joos, 1953; Smith and Hoyes, 1961; Hoffman, 1978). A wheel-type apparatus was developed to quantify the preference for the imprinting object by measuring the number of revolutions of the wheel (Johnson et al., 1985; McCabe and Horn, 1994). Recently established running wheel methods were able to automatically measure the approach distances of preference by the minute (Versace et al., 2017a,b). An automated tracking method had a time resolution of dozens of samples/second (Goldman and Wood, 2015; Lemaire et al., 2021). However, how the learning abilities for imprinting established after hatching have not been analyzed by the millisecond to investigate the relationship between the behavior and the neural activities.

Imprinting has a well-defined sensitive period (Ramsay and Hess, 1954; Hess, 1959; Bateson, 1966). In domestic chicks, the sensitive period peaks on post hatch day (Phd) 1 (Jaynes, 1957) and ends by Phd 4 (Yamaguchi et al., 2012). At Phd 4, even 2 h of training in which a chick was exposed and responded to the imprinting object did not make the chicks develop a preference for the imprinting object. The molecular mechanism of the sensitive period initiated by the inflow of the thyroid hormone into the brain has been investigated using a treadmill-type behavioral apparatus (Yamaguchi et al., 2012). In this apparatus, when chicks are close to the object on the treadmill in the training, they are forced backward to induce strong imprintability. A simultaneous choice test in the apparatus was effective in evaluating the ability to discriminate between the training and control objects because the chicks were able to observe and judge the differences between the objects with their feet firmly on the ground. Even in this treadmill-type apparatus, we could not analyze the precise timing of the chick’s response to the appearance and movements of the objects in the training. Therefore, we still have not known how to deteriorate the imprintability after the end of the period.

By improving a devise of behavioral experiment further and by categorize the learning processes in detail, we hypothesized that the entire process of imprintability in the development and deterioration will be revealed. In this study, we developed a novel disc-based behavioral apparatus and designed the training and preference tests. This new apparatus made it possible to analyze the correlation between the precise timing of the chick’s response and the appearance and movements of the objects by the millisecond. The significant improvement in the time resolution of the measurements using the disc-based behavioral apparatus allowed a clear discrimination of the different learning processes involved in the imprinting (i.e., the spontaneous approach, the acquired approach and the discrimination following approach behaviors). We examined how to develop the imprintability involved in the learning processes during the sensitive period and how to deteriorate it when the sensitive period is ending. We also examined whether the chicks trained by the disc-based apparatus showed preference to the imprinting object in the simultaneous choice test using the T-maze apparatus that we have used for many years to reveal the reliability of the novel apparatus.

## Materials and Methods

### Animals

The experiments were conducted under the guidelines of the national regulations for animal welfare in Japan, with the approval of the Committee on Animal Experiments of Teikyo University (approval number: 18-015). In this study, 106 newly hatched domestic chicks (unknown sex) of the Cobb strain (*Gallus gallus domesticus*) were used. No subjects were excluded from the analyses. Fertilized eggs were obtained from a local supplier (3-M, Aichi, Japan) and incubated at 37°C for 21 days. Chicks were hatched in the incubator in the darkness. Three to six hours after hatching, the chicks were placed in dark plastic enclosures in a breeder at 32°C to prevent light exposure till the experiments (Izawa et al., 2001). After the behavioral experiments, the animals were euthanized with an overdose of isoflurane.

### Disc-Based Apparatus for Imprinting Training

**FIGURE 1:**
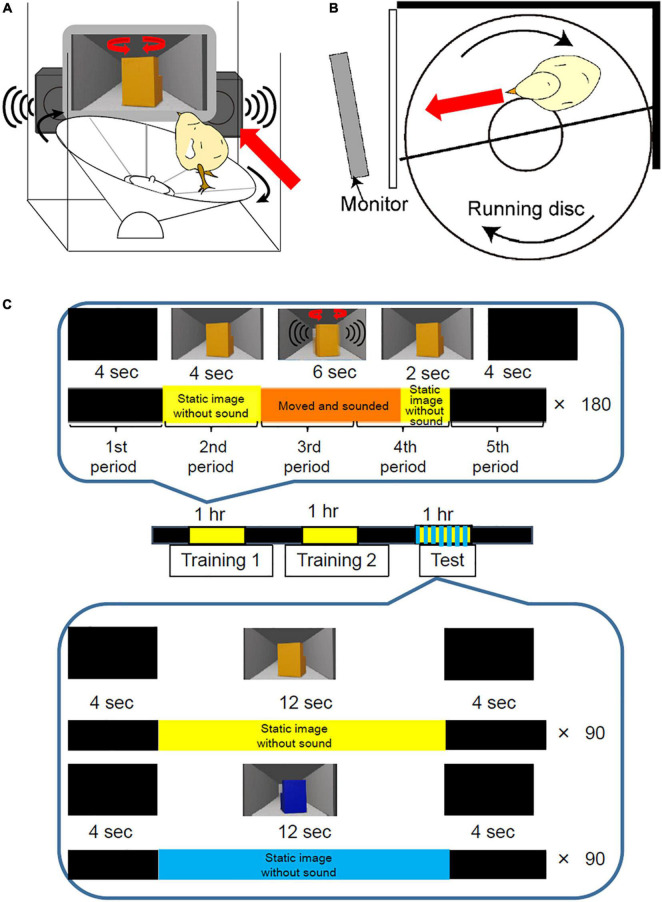
Disc-based behavioral apparatus and procedure for imprinting training and test. **(A)** The running disc-based behavioral apparatus. An imprinting object was shown through a TFT monitor which was placed in front of the apparatus. Artificial sounds were played through the speakers. **(B)** A top view of the running disc-based behavioral apparatus. The chick is surrounded by the black-colored walls made from acrylic boards or thin paper. The front wall is transparent to make the chick see the movie on the monitor. The chick represented the initial position of the behavioral experiment. **(C)** Imprinting training and testing using the disc-based apparatus. During training, two sessions were conducted at 1 h intervals. In one session of training, a movie, 20 s long, was repeatedly played on the monitor 180 times for 1 h. In the training movie, a yellow imprinting object appeared for 12 s. The object was static for the first 4 s, then turned clockwise and anti-clockwise repeatedly with artificial sounds for 6 s. After the object stopped, it remained static again for the last 2 s. During the interval, the monitor presented a black screen. To analyze the chicks’ performance during training, one trial of 20 s was divided into five periods (4 s each). In the preference test, two types of movies of 20 s were played pseudo-randomly for 1 h. In one movie, the yellow imprinting object appeared for 12 s and remained static without making a sound. In the remaining 8 s, the monitor presented a black screen. In another movie, a blue control object appeared for 12 s and remained static without sound.

We developed a new behavioral apparatus, in which the chick runs on a running disc 25 cm in diameter (Figures 1A,B and Supplementary Video 1). The chick was surrounded by the black-colored walls made from acrylic boards or thin paper which prevent a chick from dropping. The front wall was transparent to make the chick see the movie on the monitor. An imprinting object was displayed on a Thin Film Transistor (TFT) monitor (refresh rate: 56–75 Hz; size: 213 mm × 160 mm; type: LCM-T102AS, Logitec Corp., Tokyo, Japan). The distance between the apparatus and monitor was approximately 5.0 cm. Initial position of the chick was shown in Figure 1B. If the chick approached the imprinting object on the monitor, the disc rotated clockwise. After the chick approached the monitor, it was brought back to its opposite side. This apparatus sends a transistor-transistor-logic signal every 1/10 turn of the disc to a personal computer through the OmniPlex data acquisition system (Plexon Inc., Dallas, TX, United States). The sampling frequency of the recording was 2,000 samples/second. The approach distance was calculated from the recorded data of the transistor-transistor-logic signals. Since the chicks walk approximately 6.5 cm away from the center of the disc, the estimated approach distance in one lap was about 40 cm. Therefore, one transistor-transistor-logic signal was 4.0 cm approach toward the monitor by the chick.

### Imprinting Training Using Disc-Based Apparatus

For imprinting training, two sessions (training 1 and 2) were conducted at 1-h intervals (Figure 1C). One session of training involved playing a movie, which was 20 s long, repeatedly on the monitor, 180 times for 1 h. The movie was created using the 3D creation software Blender (Blender Foundation, Amsterdam, Netherlands), which has been previously used for imprinting studies on domestic chicks (Batista et al., 2016; Lemaire et al., 2021). In the training movie, a yellow imprinting object appeared for 12 s. The object was static for the first 4 s, then turned clockwise and anti-clockwise repeatedly with artificial sounds from two speakers for 6 s. After the object stopped, the static object appeared again for the last 2 s to prevent the chicks from continuously approaching after the object disappeared. The interval between the object’s appearances was 8 s. During this interval, the monitor presented a black screen. To analyze the chicks’ performance during training, a trial of 20 s was divided into five periods (4 s per period) (Figure 1C). The approach distance in each period was calculated.

### Preference Test Using Disc-Based Apparatus

We examined whether the chicks trained by the disc-based apparatus showed preference to the imprinting object in the use of the test of disc apparatus. The preference test was conducted 1 h after training 2. For the preference test using the disc apparatus, two types of movies, 20 s each, were played pseudo-randomly for 1 h (Figure 1C). In one movie, the yellow imprinting object appeared for 12 s and did not move or make a sound. In the remaining 8 s, the monitor presented a black screen. In the other movie, a blue control object appeared for 12 s and did not move or make a sound. The approach distance during the object appearing (12 s) was measured and averaged in each trial type. The preference score was calculated by “approach distance to the imprinting object/(approach distance to the imprinting object + approach distance to the control object).” If the approach distance to the imprinting object is 15 cm/trial and that to the control object is 5 cm/trial, the preference score is 0.75. If the approach distance to the imprinting object and that to the control object are exactly same, the preference score is 0.50.

### Preference Test Using a T-Maze Apparatus

For the simultaneous choice test, we used a T-maze apparatus with a main arm of 20 cm and a side arm of 69 cm (Aoki et al., 2015). A yellow imprinting object and a control object were presented through a monitor placed at both ends of the side arm of the T-maze. The objects neither moved nor made a sound. After a chick started moving from the main arm, we counted the stay time of the approach area of each object for 120 s. The test was conducted four times and the stay time for each was averaged. We then calculated a preference score by subtracting the stay time for the control object from that of the imprinting object.

### Definition of Learning Processes in Filial Imprinting

**FIGURE 2:**
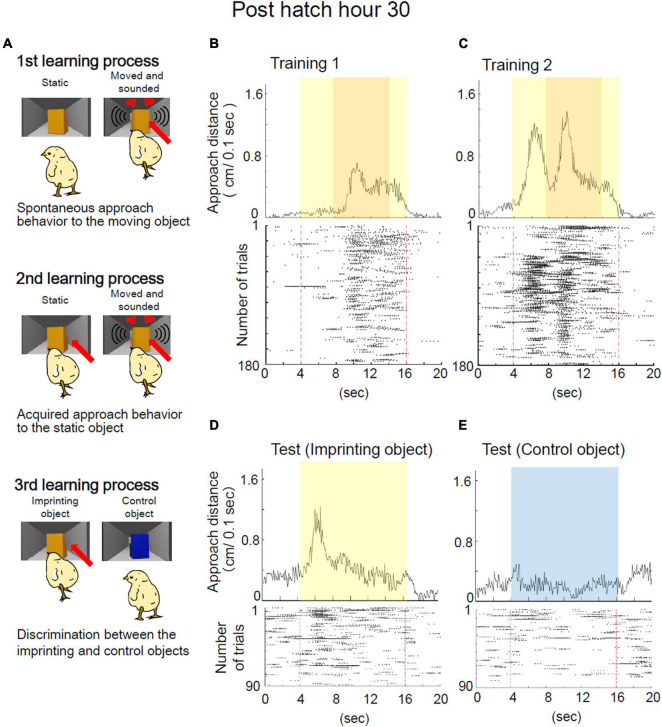
Definition of learning processes in filial imprinting. **(A)** Three learning processes for the imprinting were defined. In the first process, the chick approaches spontaneously the moving object in the training, but does not approach the static object. In the second process, the chick becomes to approach the static object in the training. In the third process, the chick discriminates the imprinting object from the control object. Representative behavior of a chick at Phh 30 in the imprinting training and test are shown in panels **(B–E)**. **(B)** Averaged approach distances in training 1 are shown in the upper column. Raster plots of each trial are shown in the lower column. One plot indicates the timing of a 1/10 turn of the disc. Red broken lines indicate the onset and offset of the object presentation. In the initial part of training 1, a chick at Phh 30 responded to the object only when it was moving. In some trials of training 1, the chick responded to the object before the object started moving. **(C)** Averaged approach distances and raster plots of each trial in training 2 are shown. In training 2, the chick responded to the static object more frequently than the chick in training 1. **(D)** Averaged approach distances and raster plots in the imprinting object trials of the test are shown. The chicks responded constantly to the static state of the yellow object. **(E)** Averaged approach distances and raster plots in the control object trials of the test are shown. The chick responded to the static state of the blue object in several initial trials, but gradually stopped responding.

The training and preference tests were designed to analyze the learning process of imprinting (Figure 1C). From the behavioral analysis, three learning processes were defined (i.e., spontaneous approach, the acquired approach, and the discrimination following approach behaviors) (Figure 2A). The representative behavior of a chick at Phh 30 is shown in Figures 2B–E. During the initial part of training 1, the chick responded to the object only when it was moving and made a sound (Figure 2B). These approaches to the object when it was moving and made sounds were considered to be a spontaneous approach behavior. This “spontaneous approach behavior” to the moving object was defined as the first learning process in imprinting. During training 2, the chick approached the static object more frequently than it did in training 1 (Figure 2C). These Approaches to the static object before it started to move as a result of exposure to repeated training trials were considered to be an acquired approach behavior. This “acquired approach behavior” was defined as the second learning process in imprinting. In the preference test, the yellow imprinting object or blue control object appeared on the monitor for 12 s, respectively. When the yellow imprinting object was presented, the chicks responded constantly to the static state of the object, as shown in the raster plots (Figure 2D). When the blue control object was presented, the chick responded to the object in some initial trials, but gradually stopped responding (Figure 2E). Accordingly, the approach distance to the imprinting object was greater than approach distance to the control object in the preference test. In this case, the preference score reached a high level (0.71). This status of the “discrimination following approach behavior” between the two objects in the test was defined as the third learning process in imprinting. The development of learning processes during the sensitive period was monitored using chicks at Phh 12, 18, 24, and 30. To examine which part of the learning processes the chicks at Phd 4 were unable to reach, the behavior of chicks at Phd 1 and 4 was compared.

### Statistical Analyses for Behavioral Data

**FIGURE 3:**
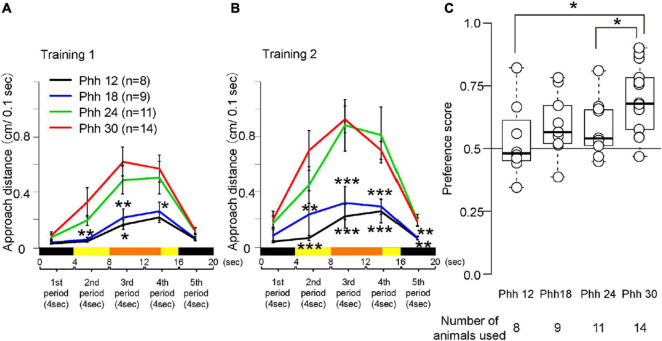
Behavior of chicks at Phh 12, 18, 24, or 30 in the imprinting training and test. **(A)** Averaged approach distances in training 1 are shown. One trial of 20 s was divided into five periods (4 s each). The black line indicates the approach distances of chicks at Phh 12. The blue line indicates the approach distances of chicks at Phh 18. The green line indicates the approach distances of chicks at Phh 24. The red line indicates the approach distances of chicks at Phh 30. The approach distances of the chicks at Phh 24 were not significantly different from those of the chicks in any period at Phh 30. The approach distances of the chicks at Phh 18 from the second to fourth period were significantly less than those of the chicks at Phh 30. The approach distances of the chicks at Phh 12 in the second and third periods were significantly less than those of the chicks at Phh 30. **(B)** Averaged approach distances in training 2 are shown. The approach distances of the chicks at Phh 24 were not significantly different from those of the chicks in any period at Phh 30. The approach distances of the chicks at Phh 12 and 18 from the second to fifth period were significantly less than those of the chicks at Phh 30. **(C)** Preference scores in the test are shown. Preference scores of chicks at Phh 12 and 24 were significantly lower than those of chicks at Phh 30. The asterisks (*) indicate significance (*p < 0.05; **p < 0.01; ***p < 0.005).

For statistical analyses, R software (R Development Core Team) or MATLAB (Mathworks, Natick, MA, United States) for Windows was used. The number of animals used is shown in Figures 3, 5 and Supplementary Figures 4–6. The equalities of variance of behavioral data were checked by the Bartlett’s test. Since the results of the Bartlett’s test showed that the variances were not equal, a Steel’s multiple comparison test or the Welch’s *t*-test was used as non-parametric tests. Using the Steel’s multiple comparison test, the behavioral data between post hatch hour (Phh) 30 and other ages were compared. We used an R script developed by Dr. Shigenobu Aoki of Gunma University^1^ to perform the Steel’s multiple comparison test. The Welch’s *t*-test was used to analyze the data and compare the behavioral data between chicks at Phd 1 and Phd 4. A paired *t*-test was used to compare the approach distance in the yellow trials with that in the blue trials in the preference test. To test the significance of the correlation between the preference score and behavioral data in the training test, Pearson’s product-moment correlation was applied. Differences were considered statistically significant at *p*-values < 0.05. The *p*-values are shown in Supplementary Tables 1–3.

**FIGURE 4:**
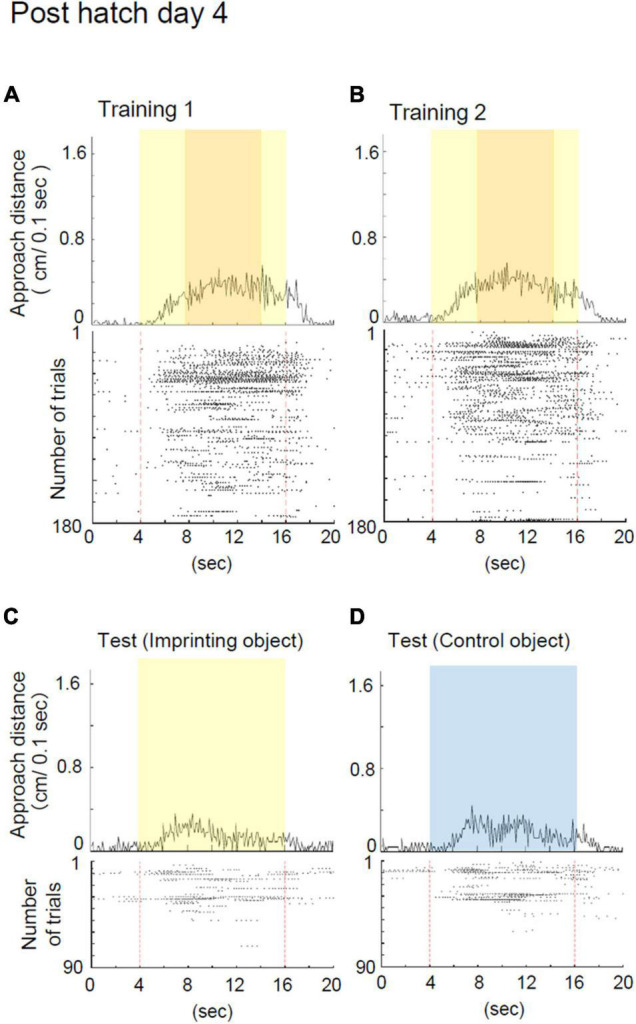
Representative behavior of a chick at Phd 4 in the imprinting training and test. **(A)** Averaged approach distances and raster plots of each trial in training 1 are shown. In training 1, the chick responded to the moving object and, in some instances, responded to the object before it started moving. **(B)** Averaged approach distances and raster plots of each trial in training 2 are shown. In training 2, the chick responded to the object in a similar manner to training 1. **(C)** Averaged approach distances and raster plots in the imprinting object trials of the test are shown. Initially, the chicks responded to the static state of the yellow object, and gradually stopped responding. **(D)** Averaged approach distances and raster plots in the control object trials of the test are shown. Initially, the chicks responded to the blue object, and gradually stopped responding.

**FIGURE 5:**
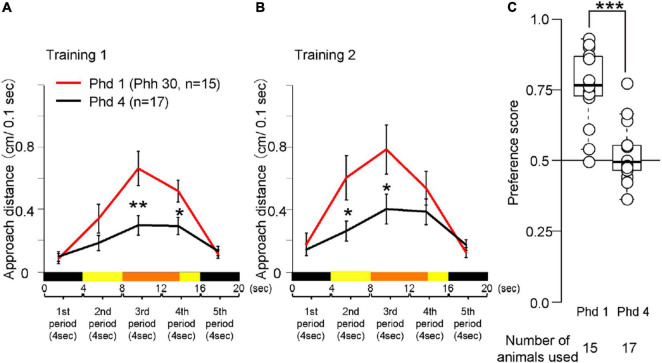
Behavior of chicks at Phd 1 and 4 in the imprinting training and test. **(A)** Averaged approach distances in training 1 are shown. The red line indicates the approach distance of Phd 1 (Phh 30). The black line indicates the approach distance of chicks at Phd 4. In the third and fourth periods, the approach distances of chicks at Phd 4 were lower than those of chicks at Phd 1. **(B)** Averaged approach distances in training 2 are shown. In the second and third period, the approach distances of chicks at Phd 4 were lower than those of chicks at Phd 1. **(C)** Preference scores in the test are shown. Preference scores of chicks at Phd 4 were significantly lower than those of chicks at Phd 1. The asterisks (*) indicate significance (*p < 0.05; **p < 0.01; ***p < 0.005).

## Results

### Establishment of Learning Processes During the Sensitive Period

The chicks at Phh 30 approached the imprinting object in the third and fourth periods when the object was moving in training 1 (Figure 3A). The chicks approached the object fewer times in the second period when the object was in a static state. In training 2, the approach distances of the chicks at Phh 30 increased from the second to the fourth period (Figure 3B). In the preference test, the chicks discriminated between the differences in the imprinting and control objects (Figure 3C). The approach distances and preference scores of the chicks at Phh 30 were compared with those of the chicks at other ages. The approach distances of the chicks at Phh 24 were not significantly different from those of the chicks in any period of training 1 and 2 at Phh 30 (Figures 3A,B). However, the preference score of the chicks at Phh 24 was significantly lower than that of the chicks at Phh 30 (Figure 3C). This means that the chicks at Phh 24 achieved up to the second learning process, but did not reach the third learning process. Subsequently, the approach distances of the chicks at Phh 12 and 18 from the second to the fifth period were significantly less than those of the chicks in training 2 at Phh 30 (Figure 3B). The preference scores of chicks at Phh 12 were significantly lower than those of chicks at Phh 30 (Figure 3C). The preference scores of the chicks at Phh 18 were not significantly different from those of chicks at Phh 30; however, they did not show clear discrimination (Figure 3C). This means that the chicks at Phh 12 and 18 achieved up to only the first learning process of imprinting.

Next, the significance of the correlations between the preference score and each period in the training trials was tested (Supplementary Figure 1A). The correlations were significant for the first to fourth periods. The results suggest that the approach to the imprinting object in training is related to the discrimination of the imprinting object in the test. The approach distances to the imprinting object will be a useful parameter of the learning process of imprinting. Furthermore, the total approach distances in training 2 were also significantly correlated with the preference score in the test (Supplementary Figure 1B). This indicates that total effort during training is also important for discrimination.

### Deterioration of Imprintability When the Sensitive Period Ended

The representative behavior of a chick at Phd 4 is shown in Figure 4. The chick at Phd 4 showed smaller responses from the second to the fourth period when the imprinting object appeared (Figure 4A). The responses continued to be smaller from the second to the fourth period even in training 2 (Figure 4B). According to the raster plots, the chick gradually stopped responding to the object in both trainings 1 and 2. In the preference test, the chick responded to the yellow object as well as the blue control object and gradually stopped responding in both trials (Figures 4C,D). In this case, the preference score was negative (0.42).

Averaged behavioral data for Phd 1 and 4 is shown in Figure 5. The behavioral data of the chicks at Phh 30 are represented as Phd 1. In training 1, the approach distances of chicks at Phd 4 were significantly less than those of chicks at Phd 1 in the third and fourth periods when the object moved and made a sound (Figure 5A). In training 2, the approach distances of the chicks at Phd 4 were significantly less than those of chicks at Phd 1 in the second and third periods (Figure 5B). In the test, the preference scores of chicks at Phd 4 were significantly lower than those of chicks at Phd 1 (Figure 5C). This suggests that the learning ability of chicks from the first learning process gradually weakened until Phd 4, such that the chicks at Phd 4 did not reach the second process of learning.

The chicks at Phd 4 showed relatively smaller responses to the imprinting object and did not discriminate it from the control object (Figure 5). This indicates that the ability to respond to moving objects was weakened so that they lost the ability to transfer the innate approach behavior to the acquired approach behavior. The scatter plots between the preference score and approach distances of the data in Figure 5 are shown in Supplementary Figures 2A,B. Interestingly, several chicks at Phd 4 still approached the imprinting object and discriminated it from the control object. This indicates that they maintained the ability of the learning process when reared in darkness until Phd 4. We also found differences in temporal changes in the behavior of the chicks at Phd 1 and 4 during the test (Supplementary Figure 3). In chicks at Phd 1, responses to the imprinting object gradually increased during the first hour of the test, whereas responses to the control object gradually decreased. In the chicks at Phd 4, both responses were less than those of chicks at Phd 1 and gradually decreased. These results suggest that both the decrease in responsiveness to the moving object and suppression of responses to the control object might cause a disability of imprinting when the sensitive period ends.

### The Simultaneous Choice Test Using the T-Maze Apparatus

In the simultaneous choice test using the T-maze apparatus, the preference scores of chicks trained by the disc-based apparatus at Phd 1 were high (Supplementary Figure 4). This indicates that chicks were able to be imprinted using the disc-based apparatus same as the previously used treadmill-type apparatus. The preference scores of chicks at Phd 1 were significantly greater than those of chicks at Phd 4 (Supplementary Figure 4), consistent to the preference score in the test using the disc-based apparatus. This suggests that the preference test using the disc-based apparatus was reliable same as the simultaneous choice test using the T-maze apparatus.

## Discussion

In this study, a disc-based behavioral apparatus was found to be advantageous for the analysis of the learning process in imprinting. In previous studies, the runway-type (Hess, 1959), wheel-type (Horn, 1986), and treadmill-type apparatus have been used for the analysis of filial imprinting (Izawa et al., 2001; Yamaguchi et al., 2012; Aoki et al., 2020). The wheel-type apparatus is advantageous while analyzing approach distances to the object because approach distances are automatically measurable (Versace et al., 2017a,b). The treadmill-type apparatus is advantageous in the reliability of imprinting acquisition because the chicks have to walk by themselves in the direction of the object through a binary choice test. In addition to those advancements above, we improved the time resolution in the disc-based behavioral apparatus. Furthermore, the training and tests were designed to define each learning process.

In previous studies using the wheel-type apparatus, the imprinting training has been performed with the physical red colored object (Johnson et al., 1985; Bolhuis et al., 1986; McCabe and Horn, 1994). In our experience, the strengths of the preference acquired by the imprinting training did not depend on the appearances of the object, e.g., physical or virtual, types of color. Using the treadmill-type apparatus, the chicks were able to be imprinted by the virtual stimulus on the monitor as well as the physical object (Takemura et al., 2018). In addition, the chicks trained with the blue-colored object showed a strong preference for the training object, similarly in the case of chicks trained with the yellow-colored object (Supplementary Figure 5). This suggests that the test was not likely to be affected by the differences in color. We think that an advantage of the virtual stimulus is that the timing of the visual and acoustic stimulus is able to be controlled exactly in every trial. That will be useful to define the learning processes in more detail. The preference scores using the novel apparatus had the same tendency of those using the T-maze apparatus (Figure 5C and Supplementary Figure 4). This suggested that the novel apparatus was available for the measurement of the preference score as well as the previous apparatus. Moreover, we are able to observe the chicks’ behavior by millisecond in the test so that the processes of the decision making may be revealed by the combinational analysis of electrophysiology.

In the learning process of imprinting, it was important that chicks responded strongly to the moving object with sounds in the first process, and become responsive to the static object without movement and sound in the second process. Pearson’s product-moment correlation between the approach distances in the second period (the static object) and those in the third period (moving object) was calculated using the data in Figures 3B, 5B. The correlation was significant in both cases (Supplementary Figures 1C, 2C). These results suggest that strong responsiveness to a moving object is necessary for the acquisition of the approach behavior toward the object in a static state.

The chicks at Phh 24 approached the imprinting object eagerly, similar to the chicks at Phh 30. However, the approach distances of the chicks at Phh 24 between the imprinting and control object trials did not differ significantly (Supplementary Figure 6). This implies that a generalization of the image occurred in the brains of the chicks at Phh 24, by which a different-colored but same-shaped object was considered the same, suggesting an immaturity of discrimination of differences of the objects. Contrastingly, in chicks at Phh 30, the approach distances in the imprinting object trials were significantly greater than those in the control object trials. This suggests that the chicks at Phh 30 showed strong preferences and imprinted on the yellow object. Moreover, they suppressed the approach behavior to the control object, suggesting a maturity of discrimination.

In this study, we developed the high time-resolution apparatus based on a running disc for the filial imprinting. Using this apparatus, we defined three learning processes for the imprinting (i.e., spontaneous approach, the acquired approach, and the discrimination following approach behaviors). We showed that the chicks at Phh 30 reached the third learning process, but the chicks before that age remained first or second learning process, and that the chicks after the sensitive period exhibited poor first learning process in the training. We propose that this apparatus is useful for the detection of the effects of infused drugs, deficits from local brain lesions, and developmental behavioral changes. In future, other statistical analyses such as generalized linear mixed models may reveal the unidentified factors which are important for the establishment of imprintability. We plan to investigate the regions of the brain involved in each learning process of imprinting by comparing gene expressions of immediate early genes among the brains at different stages of development. It is difficult to record the neuronal activity from a chick’s brain during imprinting training using the previous apparatus, especially in the case of recording from a freely moving chick. In the new behavioral apparatus, the chick will be attached with a short cord for recording on the head and will be able to run in a limited area, 24 cm in diameter, of the disc. The relationship between neural activities and the learning process of imprinting in a living chick will be clarified using a disc-based behavioral apparatus.

## Data Availability Statement

The raw data supporting the conclusions of this article will be made available by the authors, without undue reservation.

## Ethics Statement

The animal study was reviewed and approved by Animal Experiments of Teikyo University.

## Author Contributions

NA, CM, and KH were involved in the data analysis. NA and KH wrote drafts of the manuscript. All authors were involved in study design, data interpretation, critically commented and revised the drafts, and approved the final report.

## Conflict of Interest

The authors declare that the research was conducted in the absence of any commercial or financial relationships that could be construed as a potential conflict of interest.

## Publisher’s Note

All claims expressed in this article are solely those of the authors and do not necessarily represent those of their affiliated organizations, or those of the publisher, the editors and the reviewers. Any product that may be evaluated in this article, or claim that may be made by its manufacturer, is not guaranteed or endorsed by the publisher.

## Footnotes

1
http://aoki2.si.gunma-u.ac.jp/R/Steel.html

## Abbreviations

Phh: post hatch hour
Phd: post hatch day
TFT: thin film transistor.
