# Supplementary figures and images for: Imprintability of Newly Hatched Domestic Chicks on an Artificial Object: A Novel High Time-Resolution Apparatus Based on a Running Disc
Source: Front Physiol. 2022 Mar 11;13:822638. doi: 10.3389/fphys.2022.822638 (PMC8965712; doi:10.3389/fphys.2022.822638)

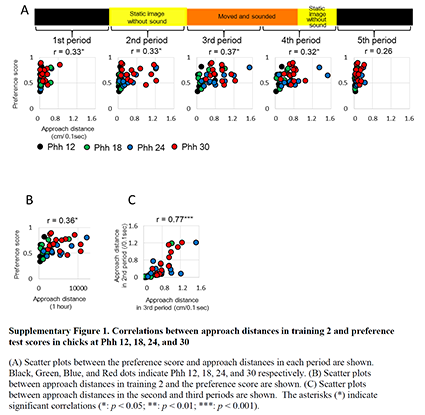

Supplement: Supplementary file 1 [file Image_1.TIF]

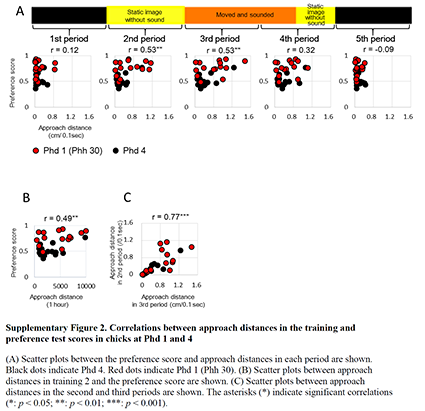

Supplement: Supplementary file 2 [file Image_2.TIF]

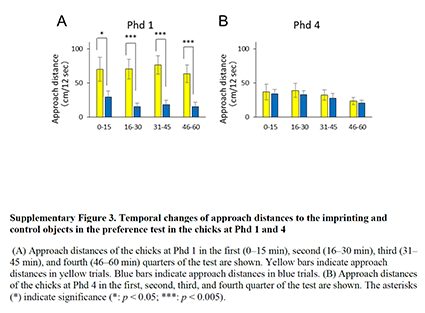

Supplement: Supplementary file 3 [file Image_3.TIF]

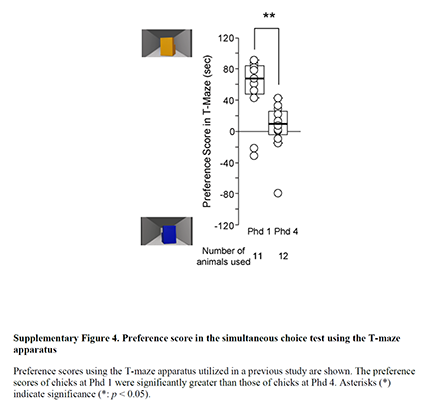

Supplement: Supplementary file 4 [file Image_4.TIF]

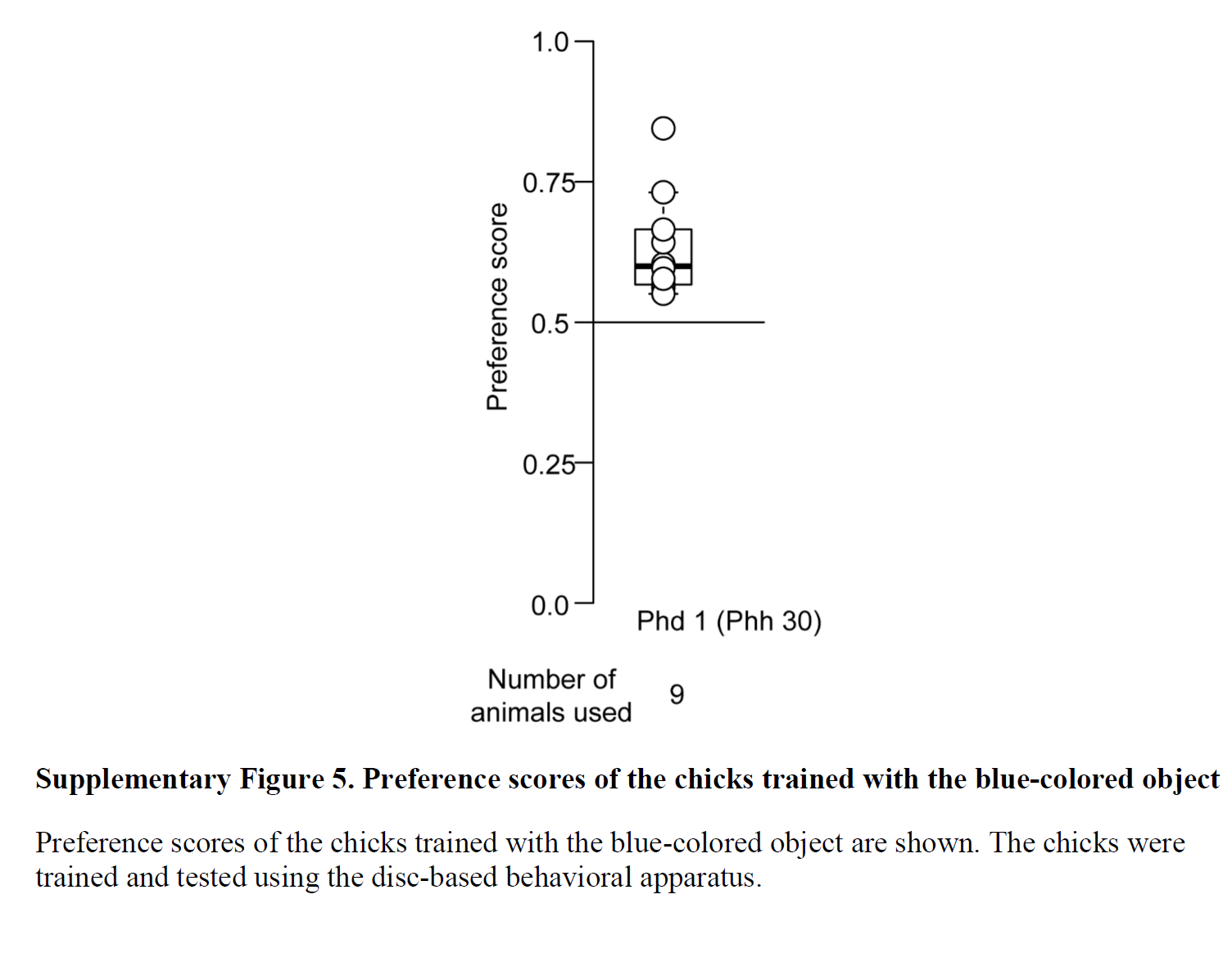

Supplement: Supplementary file 5 [file Image_5.tif]

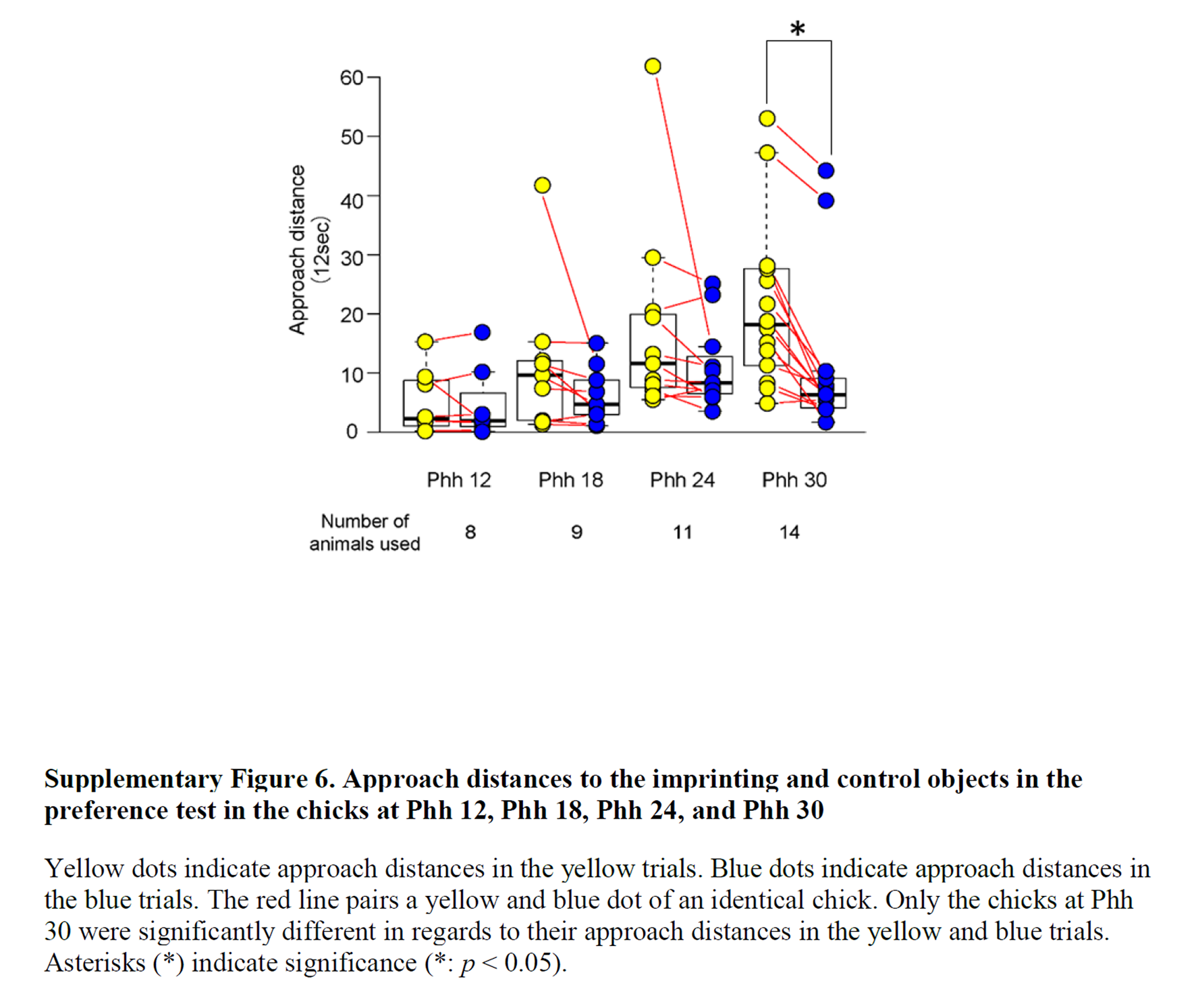

Supplement: Supplementary file 6 [file Image_6.tif]

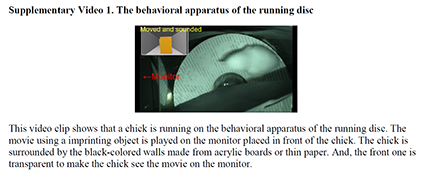

Supplement: Supplementary file 7 [file Image_7.TIF]
