# Supplementary material for: Imprintability of Newly Hatched Domestic Chicks on an Artificial Object: A Novel High Time-Resolution Apparatus Based on a Running Disc
Source: Front Physiol. 2022 Mar 11;13:822638. doi: 10.3389/fphys.2022.822638 (PMC8965712; doi:10.3389/fphys.2022.822638)
Supplement: Supplementary file 8 [file Table_1.DOCX]

| Supplementary Table 1. *p* values (Figure 3) | | |  |  |
| --- | --- | --- | --- | --- |
|  |  |  | *t* value | *p* value |
| Fig. 3A | 1st period | Phh30 vs Phh12 | 0.956 | 0.677 |
|  |  | Phh30 vs Phh18 | 2.018 | 0.116 |
|  |  | Phh30 vs Phh24 | 0.685 | 0.848 |
|  | 2nd period | Phh30 vs Phh12 | 3.071 | 0.006 |
|  |  | Phh30 vs Phh18 | 3.591 | 0.001 |
|  |  | Phh30 vs Phh24 | 0.274 | 0.988 |
|  | 3rd period | Phh30 vs Phh12 | 2.594 | 0.027 |
|  |  | Phh30 vs Phh18 | 3.213 | 0.004 |
|  |  | Phh30 vs Phh24 | 0.794 | 0.784 |
|  | 4th period | Phh30 vs Phh12 | 2.184 | 0.079 |
|  |  | Phh30 vs Phh18 | 2.709 | 0.019 |
|  |  | Phh30 vs Phh24 | 0.657 | 0.863 |
|  | 5th period | Phh30 vs Phh12 | 1.707 | 0.221 |
|  |  | Phh30 vs Phh18 | 1.828 | 0.174 |
|  |  | Phh30 vs Phh24 | 0.274 | 0.988 |
| Fig. 3B | 1st period | Phh30 vs Phh12 | 2.287 | 0.061 |
|  |  | Phh30 vs Phh18 | 2.016 | 0.116 |
|  |  | Phh30 vs Phh24 | 0.000 | 1.000 |
|  | 2nd period | Phh30 vs Phh12 | 3.686 | 0.001 |
|  |  | Phh30 vs Phh18 | 3.087 | 0.006 |
|  |  | Phh30 vs Phh24 | 0.876 | 0.731 |
|  | 3rd period | Phh30 vs Phh12 | 3.413 | 0.002 |
|  |  | Phh30 vs Phh18 | 3.150 | 0.005 |
|  |  | Phh30 vs Phh24 | 0.657 | 0.863 |
|  | 4th period | Phh30 vs Phh12 | 3.140 | 0.005 |
|  |  | Phh30 vs Phh18 | 3.402 | 0.002 |
|  |  | Phh30 vs Phh24 | 0.274 | 0.988 |
|  | 5th period | Phh30 vs Phh12 | 3.003 | 0.008 |
|  |  | Phh30 vs Phh18 | 3.024 | 0.007 |
|  |  | Phh30 vs Phh24 | 0.164 | 0.997 |
| Fig. 3C |  | Phh30 vs Phh12 | 2.389 | 0.047 |
|  |  | Phh30 vs Phh18 | 1.575 | 0.282 |
|  |  | Phh30 vs Phh24 | 2.518 | 0.033 |
