# Supplementary material for: Imprintability of Newly Hatched Domestic Chicks on an Artificial Object: A Novel High Time-Resolution Apparatus Based on a Running Disc
Source: Front Physiol. 2022 Mar 11;13:822638. doi: 10.3389/fphys.2022.822638 (PMC8965712; doi:10.3389/fphys.2022.822638)
Supplement: Supplementary file 9 [file Table_2.DOCX]

| Supplementary Table 2. *p* values (Figure 5) | | |  |
| --- | --- | --- | --- |
|  |  | *t* value | *p* value |
| Fig. 5A | 1st period | -0.296 | 0.770 |
|  | 2nd period | 1.493 | 0.150 |
|  | 3rd period | 2.858 | 0.009 |
|  | 4th period | 2.546 | 0.017 |
|  | 5th period | -0.508 | 0.615 |
| Fig. 5B | 1st period | 0.417 | 0.681 |
|  | 2nd period | 2.194 | 0.040 |
|  | 3rd period | 2.071 | 0.050 |
|  | 4th period | 1.106 | 0.279 |
|  | 5th period | -1.070 | 0.293 |
| Fig. 5C |  | 5.858 | 0.000 |
