# Supplementary material for: Imprintability of Newly Hatched Domestic Chicks on an Artificial Object: A Novel High Time-Resolution Apparatus Based on a Running Disc
Source: Front Physiol. 2022 Mar 11;13:822638. doi: 10.3389/fphys.2022.822638 (PMC8965712; doi:10.3389/fphys.2022.822638)
Supplement: Supplementary file 10 [file Table_3.DOCX]

| Supplementary Table 3. *p* values (Supplementary Figure 3-5) | | | |
| --- | --- | --- | --- |
|  |  | *t* value | *p* value |
| Supple. Fig. 3A | 0-15 min | 2.728 | 0.016 |
|  | 16-30 min | 3.986 | 0.001 |
|  | 31-45 min | 5.212 | 0.000 |
|  | 46-60 min | 4.119 | 0.001 |
| Supple. Fig. 3B | 0-15 min | 0.312 | 0.759 |
|  | 16-30 min | 0.659 | 0.519 |
|  | 31-45 min | 0.628 | 0.539 |
|  | 46-60 min | 1.127 | 0.277 |
| Supple. Fig. 4 |  | 3.129 | 0.006 |
| Supple. Fig. 5 | Phh12 | 0.418 | 0.688 |
|  | Phh18 | 1.584 | 0.152 |
|  | Phh24 | 1.481 | 0.169 |
|  | Phh30 | 6.071 | 0.000 |
